# Ethiopian *Plasmodium vivax* hypnozoites formation dynamics and their susceptibility to reference antimalarial drugs

**DOI:** 10.1186/s12879-023-08381-y

**Published:** 2023-06-13

**Authors:** Laurent Dembele, Ousmaila Diakite, Fanta Sogore, Soriya Kedir, Fatalmoudou Tandina, Mohamed Maiga, Andargie Abate, Lemu Golassa, Abdoulaye A. Djimde

**Affiliations:** 1 Université des Sciences, des Techniques et des Technologies de Bamako (USTTB), Malaria Research and Training Center (MRTC), Bamako, Mali; 2 Adama Regional Laboratory, Oromia Region Health Bureau, Adama, Ethiopia; 3 Aklilu Lemma Institute of Pathobiology, Addis Ababa University, Addis Ababa, Ethiopia; 4 College of Medicine and Health Sciences, Bahir Dar University, Bahir Dar, Ethiopia

**Keywords:** African, *Plasmodium vivax*, Hypnozoite, Invitro, Drug, Susceptibility

## Abstract

One of the key obstacles to malaria elimination is largely attributed to *Plasmodium vivax’s* ability to form resilient hypnozoites in the host liver that cause relapsing infections. As a result, interruption of *P. vivax* transmission is difficult. *P. vivax* transmission occurs in Duffy-positive individuals and have been mainly thought to be absent in Africa. However, increasing studies using molecular tools detected *P. vivax* among Duffy-negative individuals in various African countries. Studies on the African *P. vivax* has been severely limited because most of malaria control program focus mainly on falciparum malaria. In addition, there is a scarcity of laboratory infrastructures to overcome the biological obstacles posed by *P. vivax*. Herein, we established field transmission of Ethiopian *P. vivax* for routine sporozoite supply followed by liver stage infection in Mali. Furthermore, we evaluated local *P. vivax* hypnozoites and schizonts susceptibilities to reference antimalarial drugs. The study enabled the assessment of local African *P. vivax* hypnozoite production dynamics. Our data displayed the ability of the African *P. vivax* to produce hypnozoite forms *ex-vivo* at different rates per field isolate. We report that while tafenoquine (1µM) potently inhibited both hypnozoites and schizont forms; atovaquone (0.25µM) and the phosphatidylinositol-4-OH kinase (PI4K)-specific inhibitor KDU691 (0.5µM) showed no activity against hypnozoites forms. Unlike hypnozoites forms, *P. vivax* schizont stages were fully susceptible to both atovaquone (0.25µM) and the (PI4K)-specific inhibitor KDU691 (0.5µM). Together, the data revealed the importance of the local platform for further biological investigation and implementation of drug discovery program on the African *P. vivax* clinical isolates.

**Supplementary Information:**

The online version contains supplementary material available at 10.1186/s12879-023-08381-y.

## Introduction

Although *P. falciparum* causes the majority of malaria cases and deaths, *P. vivax* is the most geographically widespread human *Plasmodium* species. Outside Africa, *P. vivax* is responsible for almost half of the malaria cases where more than 2.5 billion people are at risk of infection [1] especially in the Asia Pacific region. The proportion of cases due to *P. vivax* reduced from about 8% (20.5 million) in 2000 to 2% (4.9 million) in 2021 [2]. *P. vivax* transmission is well established in southeast Asia, the Korean Peninsula to Brazil and Mexico. In Africa with most of its populations Duffy negatives, *P. vivax* infections have been increasingly reported among Duffy negative individuals [3] that were supposed to be refractory to vivax infections. Thus, *P. vivax* that appeared to be virtually absent in Africa is increasingly attracting more attention. *P. vivax* infections can result in severe symptoms and high burden of morbidity and associated mortality from profound anemia and spleen enlargement. Thus, the clinical course of *P. vivax* malaria can be both benign and severe, and needs to be considered equal to *P. falciparum* malaria regarding financial investment and life threating symptoms [4]. In Africa, malaria research focusing on *P. vivax* have been far neglected for several decades as most of malaria control program have instead focused on the deadliest *P. falciparum* infections [5]. Since the beginning of the 21st century, there has been significant success in *P. falciparum* malaria control and for the first time the antimalarial pipeline has several promising candidates (MMV: https://www.mmv.org/). However, this decline of *P. falciparum* malaria has often coincided with an increased number of *P. vivax* malaria cases [6] as most of the interventions against *P. falciparum* may not be effective for *P.vivax*. Therefore, *P. vivax* presents a barrier to malaria elimination efforts as it can form hypnozoite, a liver dormant stage that could be reactivated and cause relapses weeks to months or years after an infectious mosquito bite causing new blood-stage infections and sustaining onward disease transmission and propagation [7].

Hypnozoites have proven to be refractory to all antimalarials except for the 8-aminoquinolines of which only primaquine and the recent tafenoquine are the licensed drugs [8]. For over 50 years, the treatment of *P. vivax* has relied on a combination of chloroquine and primaquine, but this strategy is under threat. Chloroquine efficacy is now compromised [9] across many of the *P. vivax* endemic countries mainly in Africa where it has been withdrawn from many countries because of *P. falciparum* drug resistance and where exist significant operational difficulties in deploying primaquine [10] or tafenoquine [11]. Primaquine administration is associated with toxicity manifested as haemolytic anaemia and is contraindicated for individuals deficient of the glucose-6-phosphate dehydrogenase (G6PD) enzyme or pregnant women [10–12]. G6PD deficiency is a genetic disorder affecting up to 8% of the people in malaria African endemic regions [13]. As such, there is a growing need to develop new anti-hypnozoitocidal compounds to tackle this important reservoir of the parasite and prevent relapses.

However, anti-hypnozoitocidal screens entail major biological and logistical obstacles. The absence of a reliable and practical continuous cultivation system for *P. vivax* blood stages is severely limiting its access. Only some *P. vivax* field isolates are available for very few researchers. Regarding these limitations, most of compounds against malaria have originated from low to high throughput screens on the asexual blood stage of *P. falciparum.* While blood stage antimalarial assessment against *P. falciparum* can now be achieved in Africa where the medical need is the greatest and the research infrastructure, platforms are lacking, a research platform to identify hypnozoitocidal compounds and to characterize the parasite biology is much needed on the continent. In this regard, we first established field transmission of *P. vivax* for: (1) routine sporozoite supply in Ethiopia; (2) liver stage infection; (3) assessment of local *P. vivax* hypnozoite production dynamics and finally (4) evaluation of local *P. vivax* hypnozoites and schizonts susceptibilities to reference antimalarial drugs in Mali.

## Materials and methods

### Antimalarial drugs

The antimalarial drugs tafenoquine (ref: SML0396-10 mg batch: 0000029473) and atovaquone (A7986-10 mg batch: 0000042702) were obtained from SIGMA while the phosphatidylinositol-4-OH kinase (PI4K)-specific inhibitor KDU691 was obtained from Novartis [14].

### Study setting and population

The isolates were collected from febrile patients who visited health center, specifically Metehara health center, which is located in Metehara town, East Shewa Zone, Oromia Regional State, Ethiopia. *Plasmodium vivax* was reported as the most common malaria-causing species in the zone by previous studies [15, 16]. Our previous study had also documented *P. vivax* infection in Duffy negatives in the study area where both Duffy positive and negatives live side by side [17]. In this study area, earlier study showed the good performance of the assay to determine oocyst and sporozoite infection using more than 10,000 and 900 laboratory reared *Anopheles arabiensis*, respectively [18]. Those patients with self-reported febrile illness seeking malaria screening and treatment in the Metehara health center were study participants.

### Blood samples collection and processing

Using a sterile, disposable blood lancet, a finger prick blood sample was collected from each febrile patient who visited the health center for a malaria diagnosis and treatment. The thin smear was fixed with methanol and both blood smears were stained with 2% Giemsa solution. All blood films were examined by two experienced microscope reader experts blindly for each other’s findings; however, a third reader resolved the controversy results reported by the two. The *P. vivax* parasite density was calculated by averaging the results of the two independent readers. However, the Duffy blood group status of these isolates were not determined for this current ex vivo study.

### Mosquito membrane feeding assay and shipment to Bamako, Mali

Once the patients were confirmed to have *P. vivax* single infection, they were recruited to provide 5 ml of venous blood of each patient, and the sample was collected using heparin containing tube, and immediately filled to the membrane feeding glass. Membrane feeding assay was performed using colonies of *Anopheles arabiensis* that were reared in 24–27 °C temperature and humidity of 70–90% conditions in Adama insectary center as described previously [18]. At day 13 post feeding; fed live mosquitoes positive for sporozoites were shipped to Bamako, Mali using world courier service for sporozoite extraction, cell infection locally and biological assays.

#### Sporozoites source

Once mid-gut dissected, mosquitoes were observed for oocyst positivity, the remained mosquitoes of their batch were maintained for next seven (14 days post feeding) days for salivary gland dissection to ensure positivity for sporozoites and proceed shipment. Thus, *P. vivax* field isolates sporozoites were obtained from infected *Anopheles arabiensis* salivary glands collected on days 14–21 after an infective blood meal on a membrane-based feeder system at Adama malaria diagnostic center, Ethiopia as described previously [18] and the remained infected live mosquitoes shipped to Bamako, Mali, for further sporozoite isolation, hepatocytes infections (within 2 h post isolation) and further defined time point analysis. We have used 4 independent isolates of *P. vviax* in this study to generate the data. Each isolate sample was used to infect 250 mosquitoes for a total of 1000 mosquitoes and an average of 18,270; 21,540, 16,674 and 20,211 sporozoites per mosquitoes for isolates batch MT-0104, MT-0095, MT-0096 and MT-0099 that were enough to run the ex vivo assay.

#### Seeding of human primary hepatocytes

Human commercial plateable primary hepatocytes cat # HMCPTS Thermo Fisher Scientific were seeded as reported earlier [7]. Briefly: at a density of 8.3 × 10^4^ cells per well allowing cell confluence in collagenI coated µclear plate Black 96 wells (Greiner Bio-one, Ref 655,956 #1183B31 lot/ E201137Q) in complete William’s medium E (Reference: 22551-022, lot: 2,063,581,Gibco) supplemented with 10% foetal calf serum (Reference: A38400-01, lot : 2,013,379, Gibco, life technologie), 5 × 10^− 5^ M hydrocortisone hemisuccinate (Upjohn SERB Labor- atoire), 5 µg/ml Insulin (Sigma), 2 mM L-Glutamine (25030-024,Gibco), 0.02 U/ml–0.02 mg/ml Penicillin-streptomycin (15140-122, Life Technologies) and incubated at 37 °C, 5% CO2 for 24 h before infection.

#### Pre-erythrocytic cultures, assessment and drug assays

After isolation from mosquito salivary gland, the *P. vivax* sporozoites were suspended in complete William’s medium E medium and added to host’s primary hepatocyte cultures as previously reported [19]. For infection, 3 × 10^4^ sporozoites were added to each well of 96 wells plate [19]. Plates were centrifuged at 2000 rpm for 10 min at 4 °C, no break. Following centrifugation, infected plates were transferred in to incubator at 37 °C, 5% CO2 for 3 h and then washed to remove all mosquitoes debris and eventual contaminants. Then culture media are added back and renewed every 48 h. For the drug assays, the selected antimalarial drugs were added at different and known concentrations that inhibit *Plasmodium* liver stage to complete medium as follow; tafenoquine (1µM); atovaquone(0.25µM); the phosphatidylinositol-4-OH kinase (PI4K)-specific inhibitor KDU691(0.5µM) at defined times points. Infected cells were exposed to the drug for 4 days from Day 4 until fixation at Day 8 post-infection [20]. Compounds treatment was reviewed every 48 h like culture media.

Hepatic parasites were enumerated by immunofluorescence analysis [7]. Briefly, following fixation of hepatic cell and permeabilization using methanol 100%, the parasites were specifically labelled with a mouse polyclonal serum raised against the *P. falciparum* heat shock protein 70.1 (PfHSP70.1) diluted 1/1500 in 1X PBS obtained after immunization with the recombinant protein. This serum also cross-reacts with *P. vivax* HSP70 [7]. The labelled parasites were visualized with Alexa 488-conjugated goat anti-mouse immuno- globulin (Invitrogen). Parasite and host cell nuclei were stained with 1 mg/ml of diamidino-phenylindole (DAPI; Sigma). Parasites were enumerated by examination of the cultures under a fluorescence microscope at 200X magnification (Leica DM IL LED Fluo) [7]. Statistical test was done using GraphPad Prism software version 8 and t test. A *p* value *< 0.05* was considered as significant.

## Results

### ***P. vivax*** gametocytes infected mosquitos in Ethiopia produced sporozoites and different rates of hypnozoite in liver hepatocytes

**Fig. 1:**
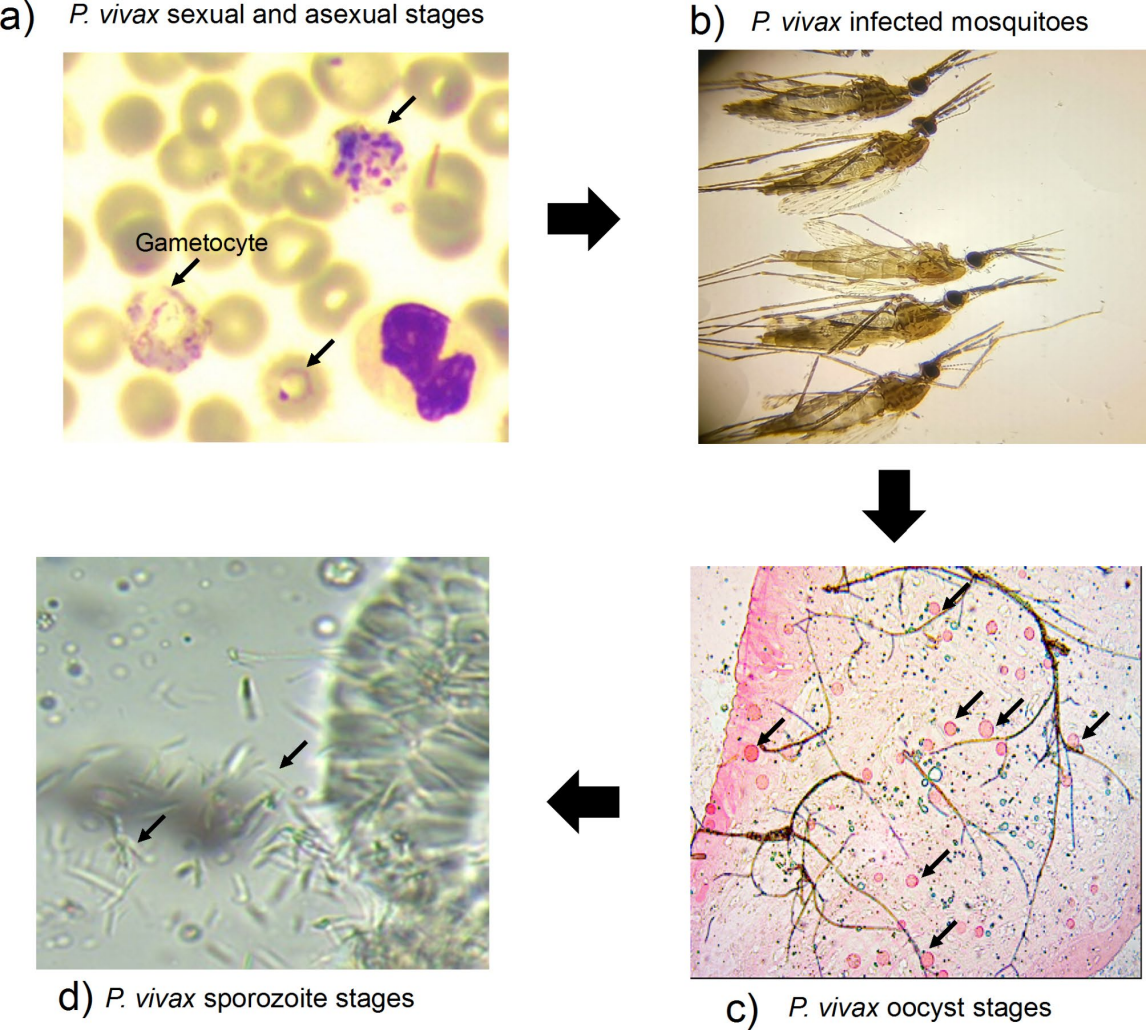
Fed mosquitoes using *P. vivax* gametocytes positive blood samples yielded oocytes and sporozoites production. **(a)***P. vivax* sexual and asexual blood stages, **(b)***P. vivax* infected mosquitoes, **(c)***P. vivax* oocyst stages and **(d)***P. vivax* sporozoite stages from salivary glands

**Fig. 2:**
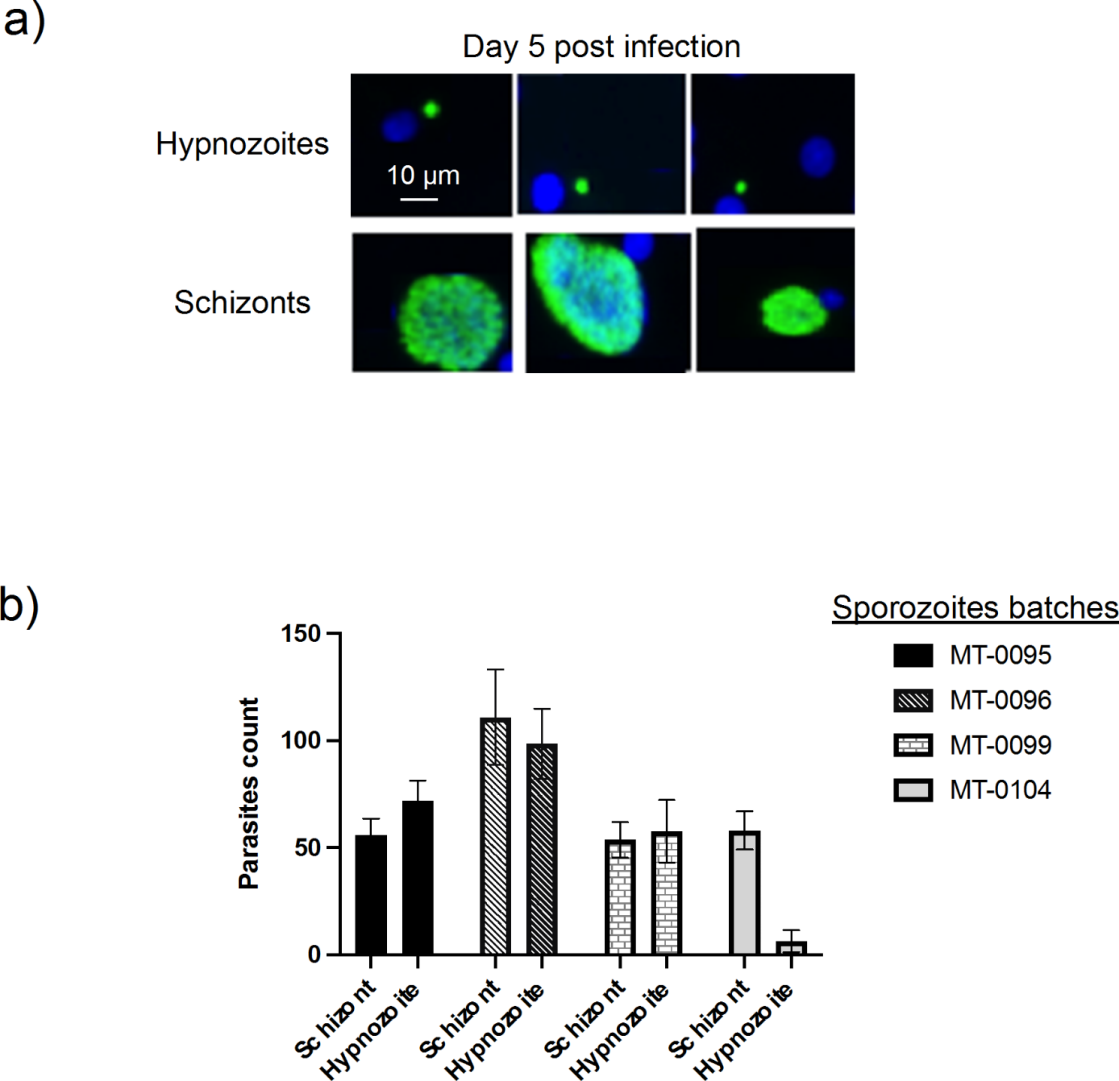
Slow growing referred as hypnozoites and normally developing pre-erythrocytic forms referred as schizonts. Two types of hepatic parasites can be clearly distinguished from days 5 post-infection, in in vitro cultured human primary hepatocytes infected with *P. vivax* sporozoites. The representative photomicrographs were made on cultures fixed on Day 5 post-infection. The parasite and host nuclei are stained with DAPI (blue), while the parasites are labelled by an antibody specific to the HSP70 of the parasite (green). **(a)** Slow growing referred as hypnozoites and normally developing pre-erythrocytic forms referred as schizonts are clearly distinguishable; **(b)** schizonts versus hypnozoites counts for different *P. vivax* field isolated parasites

We identified *P. vivax* gametocytes carriers using light microscopy (Fig. 1a). We fed over 10,000 mosquitoes to produce sporozoites (Fig. 1b, c,d). *P. vivax* gametocytes are displayed on Fig. 1a, while oocysts and sporozoites are shown on Fig. 1c and d, respectively. We selected four distinct batches of field isolated sporozoites that were used to infect the same human primary hepatocytes. We observed at day five post infection normally developing parasites referred as schizonts as well as slow developing dormant parasites referred as hypnozoite (Fig. 2a). Within the same primary hepatocytes, sporozoites batch MT-0096 displayed the greatest infectivity with higher parasite count (Fig. 2b). Sporozoites batch MT-0095, MT-0099 and MT-0104 have shown similar infectivity and parasites count (Fig. 2b). When we assessed each isolate sporozoites batch’s capability to produce dormant liver stage forms (“hypnozoites”) the hypnozoites versus schizont rate was ∼ 50/50 for batch MT-0095, MT-0096 and MT-0099 (Fig. 2b). Interestingly, MT-0104 hypnozoites count was 9-fold lower than Schizonts count (Fig. 2b). Thus, relapsing malaria parasite *P. vivax* circulating in Ethiopia can produce hypnozoite at different ratio schizont/hypnozoite. Diversity is also observed between field isolates efficiency to establish hepatocyte productive infectivity (Fig. 2).

### Differential susceptibilities to selected antimalarial drugs distinguished the two local ***P. vivax*** liver stages schizonts and hypnozoites

**Fig. 3:**
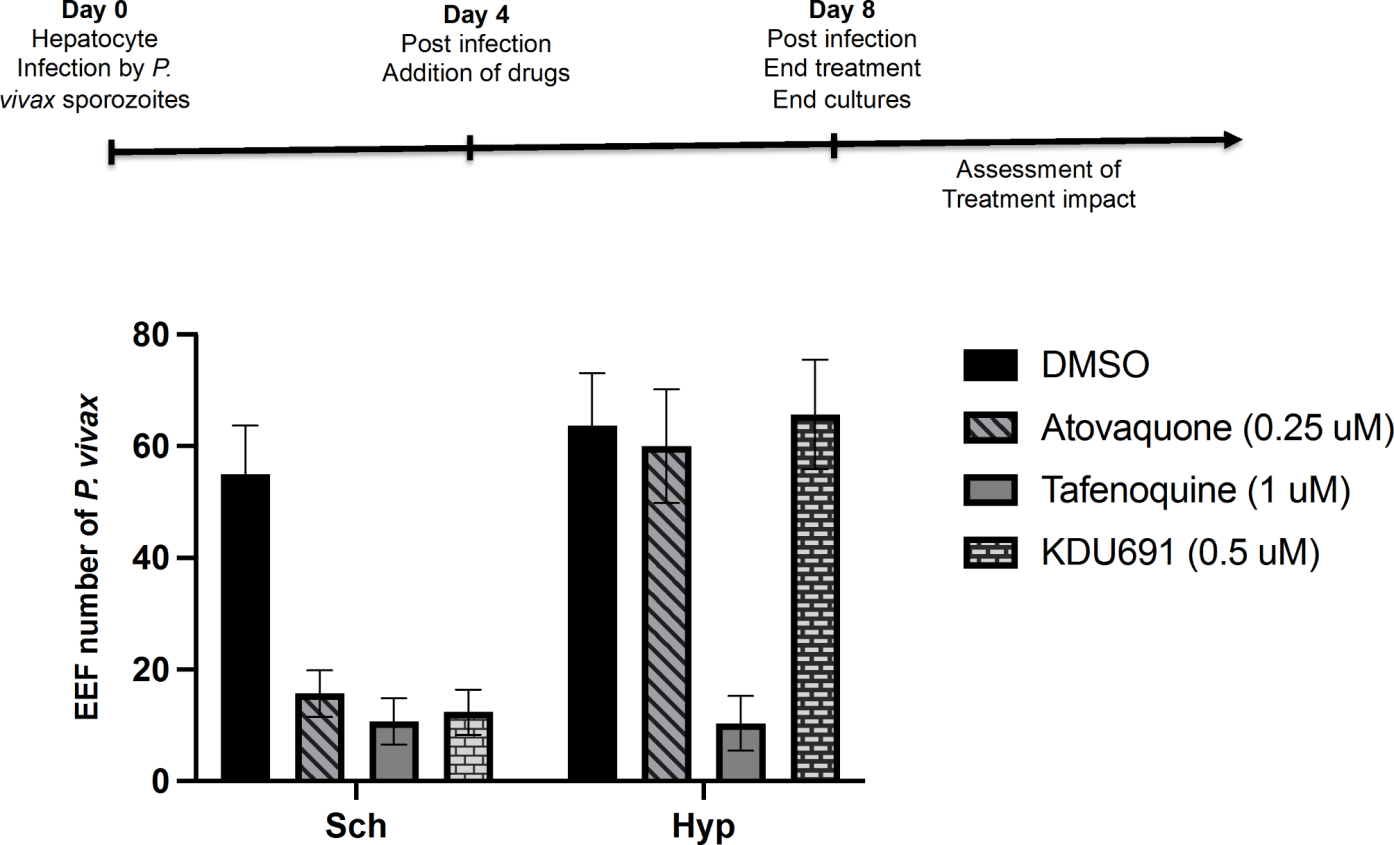
Drugs impact against distinct heptatrienes forms (schizonts versus hypnozoite) of *P. vivax* field isolated parasites. Atovaquone, tafenoquine and KDU691. DMSO is the control not treated

Having shown, that *P. vivax* circulating in Africa can produce both hypnozoite and schizont in human sporozoites infected primary hepatocytes; we set to evaluate both liver stages schizonts and hypnozoites susceptibilities to a panel of antimalarial drugs (Fig. 3). These included the atovaquone, a potent inhibitor of the parasite’s mitochondrial cytochrome bc1 complex (cyt bc1) and known to be active against *Plasmodium* liver stage schizont. Atovaquone used in the disease prevention treatment do not prevent relapse from hypnozoites. When tested against *P. vivax* liver stages, atovaquone (0.25µM) displayed potent and significant schizont inhibition (*p = 0,002*) when compared to control drug unexposed DMSO (Fig. 3). However, the dormant hypnozoites of *local P. vivax* in our culture appeared not affected by atovaquone exposure (*p = 0,67*). Like atovaquone (0.25µM); the phosphatidylinositol-4-OH kinase (PI4K)-specific inhibitor KDU691 (0.5µM) has significantly inhibited *P. vivax* liver stage schizonts (*p = 0,001*) while remained ineffective against the hypnozoites forms (*p = 0,812*) (Fig. 3). KDU691 do not prevent relapse in vivo [21]. When compound tafenoquine (1µM) known to prevent *in vivo P. vivax* relapse was used, both *P. vivax* liver stages schizonts and hypnozoites were potently inhibited, (*p = 0,0013*) and (*p = 0,0009*) respectively (Fig. 3). Thus, hypnozoites forms in our cultures displayed the expected drug metabolic susceptibility response in vitro like in vivo.

## Discussion

*P. vivax* control and elimination become very challenging because of our poor understanding of the parasite complex epidemiology, biology and the limited access to field clinical isolates. Thus, even its being the most widespread species, its continuous culture is lacking to facilitate studies. Only well-funded and equipped laboratories usually outside Africa where *P. vivax* is absent can conduct large scale studies including *P. vivax* liver stage drug discovery. Through our current efforts, we managed to setup a controlled *P. vivax* transmission in Ethiopia for regular sporozoites production (Fig. 1). As reported earlier; through this study we got good evidence of *P. vivax* malaria infection in Duffy-negative patients [22] as well as the infectivity of parasites from symptomatic *P. vivax* positive patients and their contribution for disease transmission in mosquitoes [18]. Thus, in accordance with studies outside and case reports inside Africa, *P. vivax* transmission occur in Africa and represents a threat to disease control and elimination on the continent that is still focusing only on *P. falciparum* malaria elimination.

Another interesting point, is the evidenced capability of local *P. vivax* to form dormant hypnozoites. Following investigation, we found that after five days post infection, some parasites remained small dormant [7] (Fig. 2a) in the cultures as observed in our previous studies [19]. The rate of these dormant forms was diverse between isolates (Fig. 2b) suggesting that some parasites can more productively form dormant hypnozoites. It also raises the interesting question of the parasites’ commitment to form more or less hypnozoites. Understanding this process biology is high priority as it would lead to the success of vivax malaria elimination through discovery of appropriate interventions targeting this parasite reservoir.

A further unique attribute of *P. vivax* is the absence of practical and safe treatment of vivax malaria suggesting the urgency in discovering new drugs suitable for radical cure treatment. We tested our platform suitability to screen for such compounds. Known not radical cure compounds atovaquone, a potent inhibitor of the parasite’s mitochondrial cytochrome bc1 complex (cyt bc1) and the parasite phosphatidylinositol-4-OH kinase (PI4K)-specific inhibitor KDU691 failed to potently inhibit hypnozoites forms (Fig. 3). As expected, only the 8 aminoquiline tafenoquine that prevent vivax relapses displayed strong inhibition of local *P. vivax* dormant forms (Fig. 3). The resistance of the dormant forms to both atovaquone [23] and KDU691 [21] that cannot prevent in vivo relapses of vivax malaria and their susceptibility to the radical cure compound tafenoquine [24, 25] confirmed they are likely true hypnozoites. Thus, these hypnozoites can cause relapsing malaria and disturb the phenomenon of seasonal malaria as well as disease control and elimination program strategies in Africa. Meaning that malaria would occur anytime of the year while current diverse control programs target high or low seasonal transmission window of malaria transmission. Thus, this represents a significant challenge to disease control and elimination. Our platform provides a great opportunity in Africa to screen for radical cure treatments to end vivax malaria using the circulating local parasites directly.

## Electronic supplementary material

Below is the link to the electronic supplementary material.

Supplementary Material 1: Raw data of schizonts versus hypnozoites counts for different P. vivax field isolated parasites and their susceptibility to reference antimalarial drugs

## Data Availability

All data generated are included in this published article and its supplementary information file.
